# Medicolegal aspect of loss of smell and olfactory event-related potentials

**DOI:** 10.1186/s41935-022-00306-1

**Published:** 2022-10-27

**Authors:** Cemil Çelik, Hülya Güler, Murat Pehlivan

**Affiliations:** 1 Council of Forensic Medicine, Forensic Medicine Branch Office, Kahramanmaras, Turkey; 2 Department of Forensic Medicine, Ege University Faculty of Medicine, İzmir, Turkey; 3 Department of Biophysics, Ege University Faculty of Medicine, İzmir, Turkey

**Keywords:** Odor, Clinical forensic medicine, Olfactory dysfunction, Electrophysiological test, Anosmia

## Abstract

**Background:**

It is not straightforward to objectively evaluate the olfactory dysfunction that occurs following forensic incidents. The olfactory event-related potentials method, based on electrophysiological records, may provide objective data in the evaluation of posttraumatic anosmia cases from the medicolegal perspective. This study, where a quantitative evaluation of the cases with the complaints of olfactory sensation disorder was performed using the olfactory event-related potentials test, aims to identify the factors that should be considered in the evaluation of olfactory dysfunction from the medicolegal perspective.

**Results:**

This study first evaluated the complaints of 98 patients admitted because of posttraumatic impaired smell and then administered electrophysiological odor tests on the patients. Because of this, the relationship between the EEG responses of the cases and the olfactory disorder was examined. Of the 98 cases that participated in the study, 68 (69.4%) were male and 30 (30.6%) were female. Of all cases, 53 (54.1%) had complaints of not being able to smell at all, 14 (14.3%) had complaints of reduced smell, whereas, in addition to the existing complaints of olfactory dysfunction, 44 (44.9%) of them had complaints of taste perception and 18 (18.3%) reported having vision disorders. 21 of 37 cases who reported being unable to smell during the test turned out to be anosmic. Furthermore, 16 cases stated that, though having had a response in the odor test, they had no sense of smell following the test.

**Conclusions:**

Although it seems possible to prove that there is a relationship between the olfactory event-related potential test and the diagnosis of anosmia, there is still ongoing research on its use in clinical practice. Performing both subjective and electrophysiological tests together to detect olfactory dysfunctions that occur after a forensic incident enable provide more reliable results in diagnosis.

## Background

Alongside numerous diseases and conditions, traumas are among the factors that cause olfactory dysfunction. Olfactory and gustatory dysfunctions may occur due to brain damage or olfactory nerve damage because of head and face trauma (Tai et al. 2022; Howell et al. 2018; Boesveldt et al. 2017). It can be assumed that olfactory dysfunction caused by trauma emerges because of home accidents at a rate of 26.6%, which is followed by motor vehicle accidents corresponding to the rate of 23.6% (Howell et al. 2018).

In a forensic traumatic event, the clinical picture and severity of the person need to be determined with the use of objective criteria. Due to the limitations in objectively evaluating olfactory dysfunctions there cause some difficulties in such matters as determining the severity of the dysfunction, malingering, and the relationship between the event and its cause. Moreover, there is some ambiguity about how long it takes to indicate that the olfactory dysfunction is permanent (Tai et al. 2022; Reichert and Schöpf 2018; Merabet and Pascual-Leone 2010; Joung et al. 2007).

Qualitative odor disorders incorporate parosmia (an abnormality or distortion of smell) and phantosmia (a sense of smell without a true stimulating odor) (Pellegrino et al. 2021; Hummel et al. 2022). However, given that, today, parosmia and phantosmia assessments are based on anecdotal patient experiences and limited quantitative observations due to the inability to objectively measure these disorders (Pellegrino et al. 2021). Psychophysical and electrophysiological tests are used to measure anosmia (the partial or complete loss of the sense of smell) and hyposmia (a reduced ability to smell and to detect odors), which are among quantitative olfactory disorders. Psychophysical tests are more practical and cost-effective than electrophysiological tests (Howell et al. 2018). The olfactory event-related potentials (OERP) method, as one of the electrophysiological tests, turns out to be among the most researched electrophysiological tests (Limphaibool et al.2020; Güdücü et al. 2019; Boesveldt et al. 2017).

Olfactory event-related potentials (OERP) have been investigated and subsequently considered an objective tool for measuring sensory and cognitive loss after traumatic brain injury (Invitto et al. 2018). With this method, changes in brain activity or differences can be measured in terms of bioelectrical data. This method can be clinically applied in the medicolegal evaluation of posttraumatic anosmia, because of the use of standardized stimulants and the independence of the response from the person who performs the method (Limphaibool et al. 2020; Güdücü et al. 2019; Invitto et al. 2018). However, there are still debates over the use of this method in distinguishing or diagnosing anosmic and hyposmic patients, which are of significance in medicolegal cases. (Güdücü et al. 2019). The weaknesses of the OERP method are its vulnerability to effects such as blinking, movements, and muscle activity, the requirement to keep subjects awake to have stable conditions throughout the recording session, and its response to the trigeminal nerve activity (Güdücü et al. 2019; Lötsch and Hummel 2006).

In this study, OERP test was administered on forensic cases who applied to the hospital with the complaints of post-traumatic olfactory dysfunction, and the findings of the tests were evaluated. This study aims to identify the factors that should be considered in the evaluation of olfactory dysfunction from the medicolegal perspective. It is presumed that the findings of the research will offer solutions to the primary problems in forensic reports prepared for olfactory dysfunction and thus contribute to the prevention of material and moral loss at the individual and community levels.

## Methods

### Ethics committee approval and data collection

After obtaining the Ethics Committee Approval issued, this research examined the demographic data, the types of incidents that caused the injury, the medical findings, and the olfactory test results of 98 cases with complaints of olfactory loss or reduced olfactory function due to trauma between January 1, 2017, and January 1, 2020. The participants were informed about the purpose and content of the study in detail and their consent was obtained. Those who applied for odor tests for non-traumatic reasons, those who had symptoms and complaints related to acute diseases that could block their olfactory pathways or cause olfactory complaints, and the patients whose consent could not be obtained were excluded from the research. Descriptive statistics were performed after testing the conformity of the obtained data to a normal distribution through the “IBM SPSS Statistics 25.0” program.

### Olfactory event-related potentials method

Before performing the test, each case was applied cold steam for about 30 min and they were applied decongestant (xylometazoline hydrochloride) 20 min before the test to provide a comfortable airflow in the airways. Detailed anamnesis of the patients was taken while applying cold steam to them. The negative ion generator was started working 2 to 3 h before the test to precipitate the undesirable odor particles in the air in the environment and it was kept in operation during the test. During the session, the patients were kept in a semi-reclining position under dim light and on a comfortable sofa. OERP test is a test in which the changes in electroencephalogram (EEG), caused by odorous air applied at random intervals, are recorded while the odor-free air is continuously applied to the patient’s nostril using a device called an olfactometer.

As has been observed in OERP test applications in the literature, odor-free and odorous air is delivered through a cannula placed approximately 1 cm inside the right or left nostril (Lötsch and Hummel 2006). However, this method can lead to an undesirable OERP response occurring due to stimulation of the trigeminal nerve (Lötsch and Hummel 2006). In this study, a diffuser mask (OxyMask®) was used instead of a cannula to minimize the potential of trigeminal nerve-related stimulation. The main feature of this mask is that it can deliver the air by using distributing the airflow to the nose and it has wide spaces on the side-bottom surfaces through which expiratory air can also exit (Paul et al. 2009; Ling et al. 2002).

While conducting the test via an olfactometer, the air, water, and odorant (n-amyl acetate) from the compressed tube were carried through two separate bottles at the same flow rate and the diffuser was delivered to the mask with two separate pipes. It was ensured through two pipes, one carrying an odor and the other carrying clean air, that the air was removed from the environment by means of the vacuum pump in the other room. The vacuum pump was applied to one of the two pipes through a timed solenoid valve and the undesired airflow to the mask was obstructed. Thereby, while clean air is continuously carried to the mask, it was provided to deliver the air carrying an odor for 0.6 s without changing the velocity of airflow, flow rate, or air pressure. Teflon pipes were used in the entire system to avoid a residual odor. The air was not heated and all sessions occurred at a constant temperature between 19 and 22 °C.

EEG electrodes were placed over the reference points, Fz-Cz and Cz-Pz, within the international 10/20 system. During the EEG, patients kept their eyes closed to enable eye movements not to affect the EEG monitoring. Two-channel EEG amplifier, analog-to-digital converter, driver circuits for the solenoid valve, and a computer recording system were isolated using an isolation transformer for patient safety and an independent grounding line was used. Programs were prepared with DASYLab (Graphical DAQ software) for monitoring and analysis. While this software records the monitoring process on the hard disk, it instantly displays the evoked responses on the screen.

**Fig. 1:**
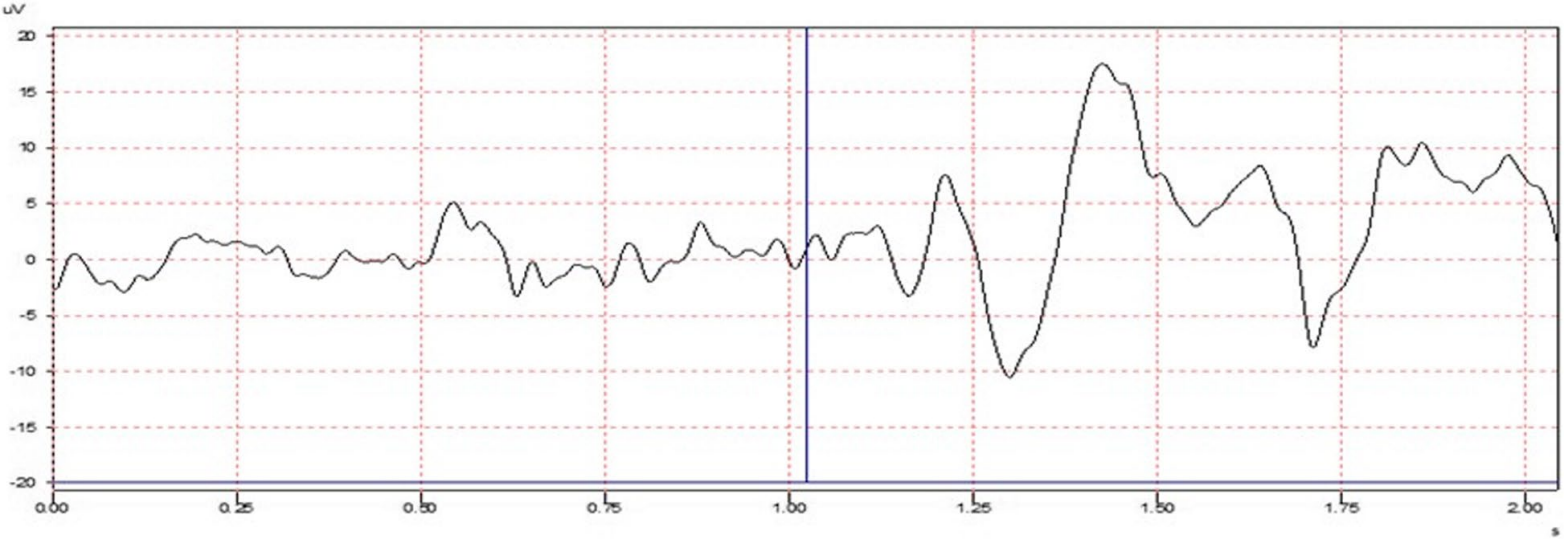
An illustration of a normal neurophysiological response to a given olfactory stimulus

An experienced observer monitored the respiratory movements of the subject and the computer screen and adjusted the system in a way to give an odor stimulant at least 10-s intervals to correspond to the beginning of the person's deep breathing. At least 3 different recordings were obtained by delivering odor stimulation to each patient simultaneously (with respiration synchronization) with the air input. 15 to 23 odor stimulants were delivered in each recording (Fig. 1 (The vertical line at the first second indicates the moment the odor stimulus is delivered)). At least two respiratory cycles were awaited while the subsequent odor stimulus was delivered so that the previous odor would move away from the respiratory cavities. During the recording, the person's respiratory type, frequency, eye movements, and, if any, the presence of any artifact in the EEG monitoring were observed.

**Fig. 2:**
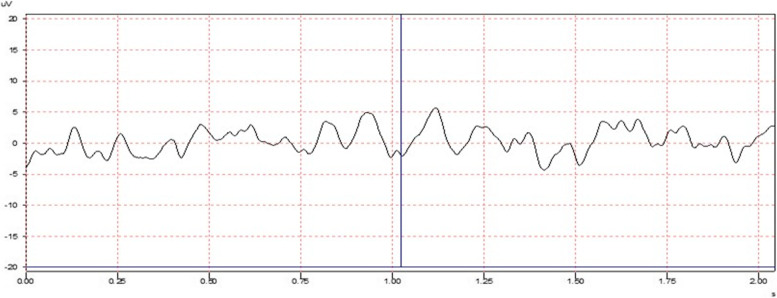
An illustration of the monitoring without a neurophysiological response to a given olfactory stimulus

In the ultimate recording, 25–50 odor stimuli were delivered to each patient with non-synchronized air input to the lungs (without the consideration of the respiratory synchronization) at random times, in other words, frequently and quantitatively enough to get used to the odor stimulation. The reason behind frequently delivering an odor is to get the subject used to the odor by adapting to a smell. Since the adaptation to the trigeminal nerve reflex was not presumed, EEG responses to olfactory stimuli were suppressed, and whether there was a false EEG response such as trigeminal activity was identified. By way of adapting to the odor stimuli, the utmost attention was paid to ensure that any stimulus other than the odor stimulus did not generate an evoked potential response (Fig. 2). After the test was administered, the patients were recorded by asking whether they sensed any odor during the test, and, if they did, they were further asked to report the time, duration, and what odor they sensed.

## Results

Sixty-eight (69.4%) of 98 cases in this study were male and 30 (30.6%) were female. The mean age of males at the date of the event was 36.01 (std. deviation: 12.68), and it was 36.46 (std. deviation: 14.82) for females. The minimum–maximum age was 12–63 in males and 14–69 in females.

Having examined the types of incidents that caused injury, it appeared that non-traffic accidents (pedestrian, etc.) corresponding to 28.6% (*n* = 28) was the leading incident. The types of incidents that caused the injury are shown in Table 1. This study also determined that 96 (98%) of 98 cases were exposed to blunt trauma to the skull during the incident (hard blow to the head, hitting the head on the floor, etc.). Two (2%) patients had a history of penetrating skull injury, without blunt trauma to the skull.

**Table 1:** Types of incidents that caused the injury

Incidents	Number (***n***)	Percentage (%)
Non-traffic accidents (pedestrian, etc.)	28	28.6
Road traffic accidents	21	21.4
Motorcycle accidents	18	18.4
Falls	11	11.2
Assault	13	13.3
Sharp object injury	5	5.1
Others (cycling accidents, occupational accident)	2	2.0
**Total amount**	**98**	**100.0**

Having examined the posttraumatic injuries, it was found that facial injuries such as fracture and soft tissue trauma occurred most frequently in 32 (32.7%) cases. They were followed by intracranial injuries such as contusion, bleeding between the cerebral membranes, and skull fracture, which occurred in 24 (24.5%) cases (Table 2).

**Table 2:** Injuries in cases with complaints of post-traumatic olfactory dysfunction

Injury location and type	Number (***n***)	Percentage (%)
Facial injury (facial fractures, facial laceration, etc.)	32	32.7
Skull fracture and intracranial injury	24	24.5
Intracranial injury	19	19.4
Skull fracture	7	7.1
Other head injuries	16	16.3
**Total amount**	**98**	**100.0**

Having examined the complaints of the cases about the olfactory dysfunction, it appeared that 53 (54.1%) applied to the hospital with the complaint of having no sense of smell and 14 (14.3%) with the complaint of reduced sense of smell (Table 3).

**Table 3:** Complaints of the cases about their olfactory dysfunction

Complaint	Number (***n***)	Percentage (%)
No sense of smell	53	54.1
Reduced sense of smell	14	14.3
The ability to notice, yet inability to detect odors	9	9.2
Inability to detect odors and reduced sense of smell	9	9.2
To perceive normal odors as not-so-normal	5	5.1
Delayed sense of smell	3	3.0
Others	5	5.1
**Total amount**	**98**	**100.0**

Information of 41 (41.8%) cases was obtained regarding when the olfactory complaints first appeared. Twenty-two (53.6%) of the 41 cases reported that their olfactory complaints started as of the incident date through the completion of the first month, whereas 18 (44%) had noticed the olfactory disorder within 1 to 6 months after the incident. It also turned out that one case’s olfactory complaints started approximately 2 years after the incident.

This research acquired information of 42 (42.9%) cases about if there was any change in their olfactory complaints over time. Of these 42 cases, 25 (59.5%) reported that there was no change, 15 (35.7%) reported recovery in their olfactory complaints over time, and 2 (4.8%) cases reported smelling unpleasant odors early days and then lost their sense of smell at all over time.

Having examined the duration between the incident and the date of OERP test, it was found that the cases applied to the hospital after an average of 44.36 (std. deviation 30.1) months following the incident. The minimum-maximum time to apply was 7–155 months.

Alongside their olfactory complaints, 44 (44.9%) cases reported having gustatory disorders, 18 (18.3%) had vision disorders, 10 (10.2%) had hearing impairment, and one case (1%) reported having tinnitus in their ears occasionally. Thirty-seven (37.8%) cases reported having amnesia. 26 (59.1%) of the 44 subjects, who reported having gustatory complaints alongside their olfactory complaints, stated that they could not taste the food as before (Table 4).

**Table 4:** Gustatory complaints

Complaints	Number (***n***)	Percentage (%)
No gustatory complaint	54	55.1
Altered taste	26	26.5
Decrease in taste sensitivity	16	16.3
Loss of taste	2	2.1
**Total amount**	**98**	**100.0**

When all OERP test results were evaluated altogether, it appeared that 75 (76.6%) cases showed a neurophysiological response to the olfactory stimulus delivered during the OERP test (Table 5). Two of the patients, who did not show a neurophysiological response in the OERP test, had applied to the hospital with the complaint of reduced sense of smell and inability to detect the smell and they then reported having no sense of smell during the test.

**Table 5:** Olfactory results of the cases as reported by the test

OERP results	Number (***n***)	Percentage (%)
Neurophysiological response in OERP	75	76.6
No neurophysiological response in OERP	23	23.4
**Total amount**	**98**	**100.0**

Thirty-seven (48.1%) of 77 cases who were asked if they smelled after the test, reported not having a sense of smell during the test, 32 (41.6%) reported having the sense of smell during the test, and 8 (10.4%) reported smelling unpleasant odors. Sixteen of the 53 patients who applied to the hospital for the complaint of having no sense of smell reported having a sense of smell during the test. Thirty-seven of the 53 cases who applied to the hospital for the complaint of having no sense of smell also reported having no sense of smell during the test. The OERP test did not show any response in 21 of the 37 cases. However, 16 of the 37 cases who reported having no sense of smell during the test had a response in the OERP monitoring. When the anamnesis, examination, medical record, and test results (no neurophysiological response in OERP) of the cases were evaluated together, 21 of the 37 cases were deemed as anosmic.

## Discussion

In this study, it appeared that the average age of males at the time of the event was 36.01 ± 12.68, and the average age of females was 36.46 ± 14.82, which is line with the systematic review and meta-analysis study by Tai et al. (2022). Considering the average age of the cases in the study, it can be surmised that the risk of their exposure to trauma is higher since they may maintain active social life at these ages. This research suggested that the reason why the number of male cases (69.4%) surpassed female cases (30.6%) is that men still tend to be more involved in work and social life than women, and they are more likely to encounter traumatic events as they tend to behave riskier than women.

Nowadays, head injuries are mostly caused by motor vehicle accidents, home accidents, cycling accidents, pedestrian accidents, and attacks, respectively (Howell et al. 2018; Schofield et al. 2014). This study revealed that 39.8% of the cases were injured in road traffic and motorcycle accidents, suggesting that motor vehicle accidents are the most common cause of injury. As this study also demonstrated, motor vehicle accidents were followed by non-traffic (pedestrian) accidents, corresponding to 28.6% (*n* = 28), and this rate appeared higher than those in the previous research.

It has been reported that the incidence of olfactory dysfunction following mild traumatic brain injury ranges from 0 to 13% and these rates can increase to 15 to 30% following moderate to severe traumatic brain injury (Tai et al. 2022). Though the loss of smell is linked to the severity of the trauma, minor trauma can also cause severe loss of smell (Tai et al. 2022; Howell et al. 2018; Boesveldt et al. 2017; Schofield et al. 2014). Similarly, this study found that 96 cases (98%) were exposed to blunt trauma to the skull during the incident, and 2 (2%) cases had nasal injuries (without exposure to head trauma) with a sharp object. In 23 (23.5%) of 98 cases exposed to head trauma, no neurophysiological response was detected as a finding supporting the diagnosis of anosmia in the OERP test. However, two cases were not evaluated as anosmic since they reported that they smelled a bit while testing.

There is a correlation between the severity of head trauma and olfactory dysfunction (Tai et al. 2022; Howell et al. 2018). This research found that facial injury (fracture, soft tissue trauma, etc.), which makes up the highest percentage, occurred in 32 (32.7%) cases, and it was followed by skull bone fracture alongside intracranial injuries (contusion, bleeding between the cerebral membranes, etc.), with 24 (24.5%) cases, and it also appeared that only intracranial injuries (contusion, bleeding between the cerebral membranes, etc.) occurred in 19 (19.4%) cases. The study is limited by the lack of information on the duration of GCS (the Glasgow Coma Scale) and post-traumatic amnesia of the patients, thus we could not contribute to the discussions in the research literature in this aspect.

It was stated that posttraumatic olfactory dysfunction is often unrecognized at the start, yet it can be noticed days or even weeks after the trauma (Tai et al. 2022; Howell et al. 2018; Schofield et al. 2014; Gudziol et al. 2014). As expected, 40 (97.5%) of the 41 cases who participated in this study reported noticing an olfactory dysfunction in the first 6 months following the trauma.

Recovery in olfactory dysfunctions is seen in approximately 20–30% of patients (Reichert and Schöpf 2018; Merabet and Pascual-Leone 2010). Though previous research stated that olfactory dysfunction most probably recovered in the first 6 months after trauma, a considerable increase in olfactory function can be expected for at least 2 years after trauma (Reichert and Schöpf 2018; Merabet and Pascual-Leone 2010; Joung et al. 2007). Approximately two-thirds (*n* = 25) of 42 cases in this study stated that there was no change in their olfactory complaints over time, and approximately one-third (*n* = 14) reported having a recovery in their olfactory complaints over time.

Epilepsy, older age, nasal polyps, diabetes mellitus, depression, hearing disorders, smoking, stroke, male gender, and upper respiratory tract infection constitute risk factors for olfactory dysfunction (Boesveldt et al. 2017; Nordin and Brämerson 2008), nowadays worthy of considered also COVID-19 infection pathologic consequences, sometimes with long-term or permanent impairment (Malta et al. 2022). Considering that the patients within the scope of this study applied after an average of 44.36 ± 30.1 months following the trauma and their duration of application varied between at least 7 months and 155 months, it appeared that the longer the application process, regardless of the legal incident, olfactory dysfunction may occur due to other risk factors such as trauma, acute or chronic diseases, aging, smoking during this process. This can also generate a causality problem in detecting the incident-related olfactory disorder. Prolonged periods in the evaluation of olfactory dysfunction make it more complicated to establish a causal relation with the incident. Applying practical odor tests to measure the success of the treatment in the early treatment process will guide the evaluation of malingering while issuing a forensic report later. Sensory loss following neurotrauma can manifest with visual, hearing, taste, and smell disturbances. Other pathologies accompanying olfactory dysfunction detected after head trauma may vary depending on the severity of the trauma and the injury area (Mehkri et al. 2022). Alongside olfactory complaints, 44.9% of the cases in this study reported gustatory disorders, 18.3% had vision disorders, 10.2% had hearing impairment, and 1% reported tinnitus. Meanwhile, 37 (37.8%) of all cases reported having a complaint of amnesia.

It is surmised that retronasal olfaction plays a key role in the perception of taste after consumption of food and liquid, and the perception perceived as flavor by the orbitofrontal cortex is formed by the combination of taste, visual, retronasal smell, and somatosensory sense of the food consumed (Mehkri et al. 2022; Xiao et al. 2021). In this study, 26 (59.1%) of the 44 cases who stated that they had gustatory complaints in addition to their olfactory disorders, reported being unable to taste the food as before. This study also revealed that, given that olfactory dysfunction affects gustatory disorders, a large part of the gustatory disorders accompanying olfactory disorders relate to taste perception. Though the loss of taste perception is an expected result of olfactory dysfunction, gustatory disorders differ from taste perception. This research revealed that in 55.1% of the cases, olfactory complaints were not accompanied by gustatory disorders.

Much previous research has evaluated the EEG response provided by the OERP test, which is particularly recommended for use in forensic cases. In this context, it was noted that the presence of OERP can distinguish more than 50% of anosmia from normosmia, and it also appeared that, while the presence of OERP suggests the availability of olfactory function, its absence has no diagnostic value (Güdücü et al. 2019). The results of this study indicated that 21 of 23 cases with no neurophysiological response (no OERP) had the complaint of having no sense of smell before the test. In 2 cases without OERP, there was a complaint of reduced olfactory perception (olfactory function is partially present). OERP was detected in 32 of 53 (54.1%) patients who applied with the complaint of having no sense of smell. OERP was identified in 75 (76.5%) of all cases. In other words, the results indicated that 75 (76.5%) cases had an olfactory function. While having olfactory function means that there is no anosmia, it does not mean that the sense of smell is normal. Olfactory function is expected in cases such as hyposmia, parosmia, and phantosmia.

Although there is clear evidence of a relationship between the psychophysical testing of smell (e.g., Sniffin Sticks Test) and OERP measurements, research for clinical applications are still ongoing (Limphaibool et al. 2020; Güdücü et al. 2019). Since there was no possibility of administering psychophysical tests on the cases in this study, this test could not be done. However, it is disclosed considering the literature review that it is possible to acquire more reliable results when psychophysical and electrophysiological tests are evaluated together in olfactory dysfunction.

Because of odor molecules transmitted throughout the room during the test, adaptation to odor occurs quickly (Xiao et al. 2021). The current research also suggests the use of a negative ion generator in the test room as a new methodological tool. A negative ion generator (air ionizer) is a device that removes contaminants from the air by ionizing air molecules and thus purifies the air (Crapser et al. 2008). During the recordings, a lower amount of odor was noticed around the negative ion generator. It is surmised that, through the use of a negative ion generator, there emerges a more sensitive EEG response to odor and more prolonged adaptation.

Thirty-seven (48.1%) of 77 cases reported not perceiving or hearing the smell delivered during the test. Although 37 cases reported not smelling following the test, 21 cases were found to be anosmic, as reported by the test, and these 21 cases reported not smelling with regard to their complaints after the test. Sixteen cases stated that although there was a response in OERP, they had no sense of smell after the test. Particularly in forensic cases, there may appear a discrepancy between the test result and the statement of the person for personal gain. Patients may report that their loss of smell occurred due to trauma, which had appeared before the event or they are likely to exaggerate their loss of smell (Limphaibool et al. 2020; Bailie et al. 2008). In such cases, OERP can disclose a possible malingering. If an EEG response is obtained to the odor stimulant delivered in the test to the patient who applied with the complaint of having no sense of smell, it is plausible to be suspected of a possible malingering. However, the fact that there is an EEG response is not an exact indicator of a normal sense of smell functioning smoothly. This is because, even if the environmental conditions are optimized, conditions such as external stimuli and blinking movements can cause an EEG response that appears as if there is a response to the olfactory stimulus. If the pressure and vacuum levels are not set equally, the trigeminal nerve can be triggered as there will be a perceptible pressure change in the nose. Such an increase in trigeminal activity may produce false results. Furthermore, even if the patient responds to the olfactory stimulant, the person may not be able to perceive the smell.

Delivering the odorous and odor-free airflow to the nostril with a cannula may cause such problems as the difficulty in keeping the cannula in the proper position, contact with the walls in the nasal passage, and air pressure changes. The trigeminal nerve activity driven by these problems may enable produce false EEG responses (Lötsch and Hummel 2006; Boesveldt et al. 2007). This study suggests that it is possible to produce more reliable results through delivering odor with a diffuser mask (OxyMask®), which is proposed as a new method by this research. The diffuser mask used in this study does not have direct contact with the epithelial tissue inside the nasal cavity. Also, there occurs no direct airflow into the nose as the airflow diffuses into the nostrils (Paul et al. 2009; Ling et al. 2002). This study surmises that erroneous EEG responses due to the trigeminal nerve activity will be minimized by using a diffuser mask.

Considerably more work will be needed to evaluate hyposmic cases with the OERP test (Güdücü et al. 2019; Lötsch and Hummel 2006). In ongoing electrophysiological studies on olfactory dysfunction, there is no marker for the degree of hyposmia. However, psychophysical tests (University of Pennsylvania Smell Identification Test or The Sniffin’ Stick Test), which enables diagnosis by scoring the olfactory threshold and show a significant correlation with each other, may act as a guideline in this regard (Limphaibool et al. 2020; Güdücü et al. 2019; Reden et al. 2016). For cases with hyposmia, parosmia, cacosmia, and phantosmia, even after concurrently evaluating the results of psychophysical tests and questionnaires measuring the quality of daily life, the assessment of olfactory dysfunctions can be left to the clinician's discretion.

## Conclusions

In the case of olfactory disorders that occur after a forensic incident, it is necessary to consider such factors as malingering, causality with an event, disorder’s permanency/temporality, the degree of olfactory dysfunction and its other effects. The OERP test recommended as an electrophysiological test turns out to be used in the diagnosis of anosmia. However, it is still unable to provide an objective evaluation for its diagnosis. Although it seems possible to prove that there is a relationship between the olfactory event-related potential test and the diagnosis of Anosmia, there is still ongoing research on its use in clinical practice. After the olfactory disorders in forensic cases became permanent, running both subjective and electrophysiological tests together will lead to more reliable results in diagnosis.

## Data Availability

The datasets used and/or analyzed during the current study are available from the corresponding author on reasonable request.

## Abbreviations

OERP: Olfactory event-related potentials
EEG: Electroencephalogram
